# Evaluation of rs10811661 polymorphism in CDKN2A / B in colon and gastric cancer

**DOI:** 10.1186/s12885-023-11461-6

**Published:** 2023-10-16

**Authors:** Maria Beihaghi, Reza Sahebi, Mohammad Reza Beihaghi, Raheleh Khosravi Nessiani, Majedeh Ramian Yarasmi, Sajad Gholamalizadeh, Fatemeh Shahabnavaie, Mitra shojaei

**Affiliations:** 1 Department of Biology, Kavian Institute of Higher Education, Mashhad, Iran; 2 School of Science and Technology, The University of Georgia, Tbilisi, Georgia; 3 Department of Modern Sciences and Technologies, Faculty of Medicine, Mashhad University of Medical Sciences, Mashhad, Iran; 4 Department of Public Health, Sheffield Hallam University, Sheffield, South Yorkshire England; 5 Department of Chemical Engineering, Sahand University of Technology, Tabriz, Iran; 6 Department of Biotechnology, Ferdowsi University of Mashhad, Mashhad, Iran; 7 Faculty of Science, Ferdowsi University of Mashhad, Mashhad, Iran

**Keywords:** Colon cancer, CDKN2A / B gene, Polymorphism rs10811661, Statistical analysis, RFLP-PCR, Gastric Cancer, Biomarker

## Abstract

One of the causes of colon and gastric cancer is the dysregulation of carcinogenic genes, tumor inhibitors, and micro-RNA. The purpose of this study is to apply rs10811661 polymorphism in CDKN2A /B gene as an effective biomarker of colon cancer and early detection of gastric cancer. As a result,400 blood samples, inclusive of 200 samples from healthy individuals and 200 samples (100 samples from intestinal cancer,100 samples from stomach cancer) from the blood of someone with these cancers, to determine the genotype of genes in healthful and ill people through PCR-RFLP approach and Allelic and genotypic tests of SPSS software. To observe the connection between gastric cancer and bowel cancer risk and genotypes, the t-student test for quantitative variables and Pearson distribution for qualitative variables have been tested and the results have been evaluated using the Chi-square test. The effects confirmed that the highest frequency of TT genotypes is in affected individuals and CC genotype is in healthful individuals. In addition, it confirmed that women were more inclined than men to T3 tumor invasion and most grade II and III colon cancers, and in older sufferers with gastric cancer, the grade of tumor tended to be grade I. Among genetic variety and rs10811661, with invasiveness, there is a tumor size and degree in the affected person. In summary, our findings suggest that the rs10811661 polymorphism of the CDKN2A / B gene is strongly associated with the occurrence of intestinal cancer and stomach is linked to its potential role as a prognostic biomarker for the management of bowel cancer and stomach.

**Supplementary Information:**

The online version contains supplementary material available at 10.1186/s12885-023-11461-6.

## Introduction

Gastric cancer (GC) is the fifth most common cancer and the third main cause of cancer deaths, accounting for about 800,000 deaths annually [1]. In popular, the common age of onset is 60 to eighty years, and under 30 years is rare. For this reason, GC is taken into consideration as an aging sickness [2, 3]. The countries of East Asia, accompanied by Eastern Europe and South America, have the highest rates of infection. The lowest rates are observed in North America and Africa. However, the prevalence of GC has decreased in most countries, and regardless of advances in diagnosis, the ailment is normally recognized at advanced levels, in particular, because of the nonspecific nature of the signs within the early ranges. The average 5-year survival is only 20%. Further, surgical treatment and chemotherapy are of restricted value in treating advanced instances. Further, a wide variety of centered cures are available using molecular markers [2].

GC is a multifactorial sickness that includes a way of life, aging, socioeconomic factors, infectious agents inclusive of Helicobacter pylori (classified as group 1 carcinogenic and associated with 80% of cases), Epstein-Barr virus (related to 10% of tumors), and multiple genetic and epigenetic versions [3, 4]. Consistent with Loren’s type, gastric adenocarcinomas are divided into types: Intestinal (nicely differentiated with cohesive neoplastic cells, forming tubular gland-like systems) and diffuse (poorly differentiated with the aid of penetration and thickening of the gastric wall without discrete loads) [5]. Those two kinds fluctuate now not handiest in histological evaluation, but additionally in sex, age, and other epidemiological functions [2, 6, 7].

Colorectal cancer is the third most common tumor and the fourth leading cause of demise within the eastern world [8]. The prevalence of this cancer varies from race to race; it is also related to age, diet, family history, smoking, sedentary lifestyle, and alcohol consumption [9]. Another cause of colorectal cancer is polyps. Polyps are clusters of cells that develop within the middle of the colon. These polyps may transform into malignant tumors [10]. Further to many of these factors, the function of genetic factors in the spread of colon cancer can’t be disregarded. One of the pathways in which the polymorphism of the involved genes is related to colorectal cancer is the insulin-signaling pathway [11]. Insulin is a hormone secreted by beta cells inside the islets of the pancreas. This hormone’s function in regulating blood sugar (glucose) is known. Insulin controls cellular growth with the aid of binding to its receptor [12] and prevents the deliberate loss of life of cells [13]. From the factor of view of molecular biology, the insulin receptor is a membrane receptor that is activated by binding to insulin [14]. After the insulin binds to its receptor, the subunits of the insulin receptor are phosphorylated and the insulin-signaling pathway for glycogen formation is decreased [15]. Due to the fact tyrosine kinase receptors are involved in programmed cell death, metastasis, and cell growth and proliferation, their effect on malignancies isn’t unexpected [16].

In place 5, there is an area of insulin receptor genes that is wealthy in GC, and transcription factors are attached to it [17]. Polymorphism in this area causes a decrease in the degree of insulin receptor expression [18, 19]. Numerous genetically related studies have shown that genetic changes on chromosome 9p21 can be concerned in numerous malignancies which include leukemia, glioma, ovarian, breast, and pancreatic cancers [20–26]. This location is understood for the cyclin A and B-established kinase inhibitors called CDKN2A / B, which are involved in numerous metabolic and pathological issues consisting of diabetes, metabolic syndrome, cardiovascular disease, and Alzheimer’s [27–30].

Current data have proven that the CDKN2A / B gene can alter cell increase by using stopping the cell cycle in section G1. Cell cycle progression inside the G1 phase is mainly modulated through the p14ARF, p15INK4B, and 16INK4A proteins ( [27, 31, 32]). Tumor suppressor proteins p15INK4B and 16INK4A stop the cell cycle by decreasing cyclin-dependent kinases 4 and six (CDK4, 6), whilst p14ARF protein promotes apoptosis and mobile cycle by promoting the m5m2 -p signaling pathway through p5 mdmart [33, 34]. Whilst the tumor suppressor’s p14ARF and p16INK4A are proven to be encoded by CDKN2A, the p15INK4B protein is encoded using CDKN2B [34]. There’s new evidence that genes inside the CDKN2A / B locus genes were mutated or deleted in several human cancers. Several SNPs at the CDKN2A / 2B site lessen their expression and result in tumor cell proliferation and progression [30, 35]. Current data have proven that deletion of CDKN2A / B is associated with poor prognosis and lower survival in patients with cutaneous T mobile lymphoma [36]. In another large-scale meta-analysis study performed by Lou et al., the relationship between CDKN2A / B rs4977756 gene polymorphism and the risk of glioma in 18,893 patients without or with cancer was investigated. This evaluation showed that rs4977756 polymorphism is drastically related to the risk of glioma [37]. Consistent with that research, the polymorphism correlation of the CDKN2A / B gene (rs10811661) was studied in 564 breast cancer sufferers and the outcomes confirmed that people with TT genotype have been extra susceptible to breast cancer [38]. The affiliation of the two SNPs changed into assessment with the aid of CDKN2A / B locus (rs1333049 and rs10811661) and the clinical manifestations of esophageal squamous cell carcinoma (ESCC) and counseled that the CC rs1333049 genotype was polymorphically associated with a weaker overall prognosis [39]. the aim of this study was to assess the association between the CDKN2A / B rs10811661 polymorphism, tumor grade and tumor invasiveness in individuals with colon and gastric cancer.

## Materials and methods

### Patient samples

100 fresh colon tumors, 100 fresh stomach tumors, and 200 non-tumor tissue biopsies were obtained from the Biobank of the Research Center for Gastroenterology and Hepatology (Imam Khomeini Medical Institute, Tehran). Instances of colorectal and belly cancers have been identified with colonoscopic findings and then histopathological evaluation. Also, histopathological features were scored according to the American Joint Committee on Cancer Criteria and the TNMG Procedure for Classification of Stage and Grade. The whole experimental procedure was approved by the Ethics Committee of Mashhad University with (IR.MUMS.MEDICAL.REC.1397.468) approval number. Written informed consent has been obtained from all patients for the study, prior to surgical removal of their tumors.

### DNA genotype

Peripheral blood samples had been taken from all patients and controls for genetic exams. Genomic DNA became extracted from peripheral blood with the usage of the standard phenol-chloroform technique [40]. The sequence of BSPHI polymorphism (rs10811661) using approach PCR (Polymerase chain response) unique primers forward: 5’-cagaatggtcttaaaaatgggtgt3’, Reveres: 5’-ttcagaataagaaacaaggcaac3 ' was performed. The primers were designed using gene sequences obtained from Gene Runner and Primer3 websites and software received from NCBI. Conditions and PCR application have been as follows: first 5 mins of initial denaturation at 95 ° C and then 35 cycles with this application 32 seconds of denaturation at 95 ° C, 42 seconds at 62 ° C to connect the primers, 42 seconds were executed at 72 ° C for propagation and on the end of 5 minutes at 72 ° C for final propagation. PCR products have been digested using BSPHI restrict enzyme (fermentas) at a reaction rate of 5U for 16 hours at 37 ° C. due to the truth that for this enzyme, in the presence of an ordinary (pro) allele, there may be a cut-off site in the Reproduction part. Analysis of all RFLP products become performed on 2% agarose gel and the gels have been stained using Greenview.

### Statistics

For statistical analysis of the received information, SPSS statistical software program was used. The Hardy-Weinberg equilibrium and the Pearson distribution were used to evaluate the allelic and genotypic frequency of the CDKN2A / B gene in rs10811661 polymorphism. The connection between gastric and intestinal most cancers danger and genotypes become tested using a student’s t-test for quantitative variables and Pearson distribution for qualitative variables. The obtained outcomes have been evaluated with the use of the Chi-square statistical method and considering the P-value less than 0.05 as a different significance.

## Results

### Identification of rs10811661 genotype in cdkn2a / b gene

**Fig. 1:**
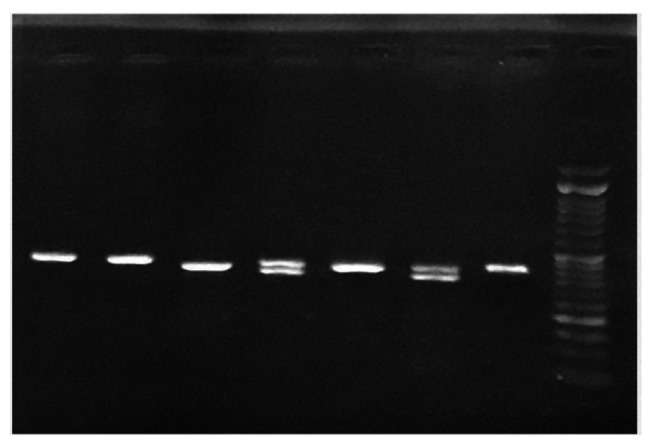
The first column on the right is for DNA-sized markers. The second column is related to the homozygous genotype (CC) with a 393 bp bond. The third column represents the homozygous genotype (TT) with 344 and 49 bp bonds

The genotypes were determined in such a way that in individuals with homozygous genotype (TT), two pieces of RFLP (Restriction Fragment Length Polymorphism) products with lengths of 344 bp and 49 bp were formed. In homozygous (CC) individuals who lacked a shear site. One piece of 393 bp was observed and in heterozygous individuals (CT), three pieces (393, 344, 49) were obtained in Fig. (1).

### Investigation of frequency in the studied conditions

The following were found in colorectal cancer according to Table (S1) in supplementary file:

1. The frequency of different rs10811661 polymorphisms in the CDKN2A / B gene was studied and CC (19%), CT (40%), and TT (41%) were determined.
2. The frequency of the subjects according to age is 17% under 30 years, 40% (31–40), 36% (41–50), and 7% (51–60).
3. Frequency tests are based on the stage of tumor growth, stage I (26%), stage II (50%), and stage III (24%).
4. Evaluation of tumor invasiveness, invasive stage T3 (46%), highest frequency, and T2 (32%) and T1 (22%) were observed.

The results showed the highest frequencies are in the TT genotype and age (40 − 31), in grade II, and in terms of invasiveness in the T3 stage.

The following were found in gastric cancer according to Table (S2):

1. The frequency of different rs10811661 polymorphisms in the CDKN2A / B gene was studied and CC (19%), CT (40%), and TT (41%) were determined.
2. The frequency of the subjects according to age is 31% under (25–34), 37% (35–43), and 32% (44–52).
3. Frequency tests are based on the stage of tumor growth, stage I (32%), stage II (37%), and stage III (31%).
4. Evaluation of tumor invasiveness, invasive stage T3 (46%), highest frequency, and T2 (32%) and T1 (22%) were observed.

The results showed that the highest frequency in TT genotype is in age (43 − 35), in grade II, and in terms of invasion in the T3 stage.

Examining colon and stomach cancers, we came to the common conclusion that both have the highest frequency in the age range (30–45), in the TT genotype, in the T3 stage, and in II degrees.

### Bilateral frequency

#### Study investigating the relationship between age and variables in colon cancer

In the study of the relationship between age and variables, the following items were found according to Table (S3, S4, S5):

1. In examining the relationship between age and genotype according to Table (S3), no significant level was obtained for Spearman correlation, i.e., the correlation rate was more than 0.05 and equal to 0.232, so with a confidence of 0.95 statistical assumptions of zero relationships between age and Genotype rejected Spearman correlation value is -0.123.

In the analysis of variance according to Table (S4) between the relationship between age and genotype, the value of the F statistic is equal to 0.837 and the probability value related to its significance is equal to 0.436, which is more than 0.05, so with confidence, 0.95. The statistical null hypothesis that the mean age is equal to the genotypes is confirmed, so we can say that there is no significant difference between the mean groups.

Regarding the means from the highest to the lowest, they are related to CC, CT, and TT, respectively, and their values are equal to 40.7368, 39.62,5, and 38.122, respectively.

1. In examining the relationship between age and tumor invasiveness according to Table (S3), based on Spearman correlation, it is more than 0.05 and equal to 0.955, so with a confidence of 0.95 statistical assumptions of zero relationships between age and tumor invasiveness rejected Spearman correlation value is -0.006.

In the analysis of variance according to Table (S4), the relationship between age and tumor invasiveness, the value of the F statistic is 0.058 and the probability value of its significance is equal to 0.944, which is more than 0.05, so with 95% confidence. The statistically zero hypothesis that the mean age is equal to the invasiveness of the tumor is confirmed, so it can be said that there is no significant difference between the mean of the groups.

Regarding the means from the highest to the lowest, respectively, related to T1, T3, and T2, their values are equal to 39.5, 39.3478, and 38.8438, respectively.

1. In the study between age and tumor stage, a significant level was not obtained for Spearman correlation, i.e. the correlation was more than 0.05 and equal to 0.232, so with 0.95 confidence, the statistically zero hypotheses that there is a relationship between tumor stage and age is rejected. The value of the Spearman correlation is equal to -0.12.

In the analysis of variance comparing the mean age and stage of the tumor, the value of the F statistic is 0.767 and the probability value of its significance is equal to 0.467, which is more than 0.05, so with 0.95 confidence, the statistical assumption of zero based on the mean age is confirmed to be equal to the tumor stage, so it can be said that there is no significant difference between the mean groups.

The means from highest to lowest are related to stage I, stage II, and stage III, respectively, and their values are equal to 40.5769, 39.16, and 37.875, respectively.

In examining the relationship between age and variables in colon cancer, the highest mean includes CC genotype (40.7368) in invasive state T1 (39.5) and stage I (40.5769).

#### Study investigating the relationship between age and variables in gastric cancer

In the study of the relationship between age and variables, the following items were found according to Table (S6, S7, S8):

1. In examining the relationship between age and genotype according to Table (S6), no significant level was obtained for Spearman correlation, i.e. the correlation rate was more than 0.05 and equal to 0.223, so with a confidence of 0.95 statistical assumptions of zero relationships between age and Genotype rejected Spearman correlation value is -0.123.

In the analysis of variance according to Table (S7) between the relationship between age and genotype, the value of the F statistic is equal to 0.837 and the probability value related to its significance is equal to 0.436, which is more than 0.05, so with confidence, 0.95The statistical null hypothesis that the mean age is equal to the genotypes is confirmed, so we can say that there is no significant difference between the mean groups.

Regarding the means from the highest to the lowest, they are related to CC, CT, and TT, respectively, and their values are equal to 40.7368, 39.625, and 38.122, respectively.

1. In examining the relationship between age and tumor invasiveness according to Table (S6), based on Spearman correlation, it is more than 0.05 and equal to 0.285, so with a confidence of 0.95 statistical assumptions of zero relationships between age and tumor invasiveness rejected Spearman correlation value is 0.108.

In the analysis of variance according to Table (S7), the relationship between age and tumor invasiveness, the value of the F statistic is 0.058 and the probability value of its significance is equal to 0.944, which is more than 0.05, so with 95% confidence. The statistically zero hypothesis that the mean age is equal to the invasiveness of the tumor is confirmed, so it can be said that there is no significant difference between the mean of the groups.

Regarding the means from the highest to the lowest, respectively, related to T1, T3, and T2, their values are equal to 39.5, 39.3478, and 38.8438, respectively.

1. In the study between age and tumor stage, a significant level was not obtained for Spearman correlation, i.e. the correlation was more than 0.05 and equal to 0.755, so with 0.95 confidence, the statistically zero hypotheses that there is a relationship between tumor stage and age is rejected. The value of the Spearman correlation is equal to -0.32.

In the analysis of variance comparing the mean age and stage of the tumor, the value of the F statistic is 0.767 and the probability value of its significance is equal to 0.467, which is more than 0.05, so with 0.95 confidence, the statistical assumption of zero based on the mean age is confirmed to be equal to the tumor stage, so it can be said that there is no significant difference between the mean groups.

The means from highest to lowest are related to stage I, stage II, and stage III, respectively, and their values are equal to 40.5769, 39.16, and 37.875, respectively.

In examining the relationship between age and variables in colon cancer, the highest mean includes CC genotype (40.7368) in invasive state T1 (39.5) and stage I (40.5769).

The study of colon and gastric cancers both contained the highest values in CC genotype (40.7368) in invasive state T1 (39.5) and stage I (40.5769).

#### Investigating the relationship between genotype and variables in colon cancer

In examining the relationship between genotype and the studied variables, the following items were found according to Table (S9, S10, S11):

1. The highest frequency of the CC genotype is in the stage II group, the highest frequency of the CT genotype is in stage III and the highest frequency of the TT genotype is in stage II.

The value of the Chi-square-Pearson statistic is equal to 2.63 and its significance level is 0.638, which is more than 0.05, so with 0.95 confidence, the statistical null hypothesis of no relationship between tumor stage and genotype is confirmed. Thus, there is no relationship between tumor stage and genotype.

Regarding the results of the equality test, the mean of the tumor stage component based on the genotype has a chi-square value of 0.447 and the probability value related to its significance is equal to 0.8, which is greater than 0.05, so with a confidence of 0.95, the null hypothesis is zero Statistics confirm that the means are equal.

1. The highest frequency of tumor invasion is related to the CC genotype in the T2 group and the lowest rate is related to the T1 group.

The value of the Chi-square-Pearson statistic is equal to 1.539 and its significance level is 0.836, which is more than 0.05, so with the confidence of 0.95, the Statistical null hypothesis that there is no relationship between tumor invasion and genotype is confirmed, so there is no relationship between tumor invasion and genotype.

Regarding the results of the equality test, the mean component of the tumor’s invasiveness based on genotype has a value of 1.461 and the probability value related to its significance is equal to 0.482, which is more than 0.05, so with a confidence of 0.95, the null hypothesis Statistics on the equality of means are confirmed.

#### Investigating the relationship between genotype and variables in gastric cancer

In examining the relationship between genotype and variables, the following items were found according to Table (S12):

1. The highest frequency of the CC genotype is in the stage I group, the highest frequency of the CT genotype is in stage II and the highest frequency of the TT genotype is in stage III.
2. The highest frequency of tumor invasion is related to the CC genotype in the T3 group and the lowest rate is related to the T1 group.

In the study of colorectal cancer, the relationship between genotype and variables in the relationship between the two diseases, in colon cancer, the highest frequency of CC genotype in group II, the highest frequency of CT genotype in stage III, and the highest frequency of TT genotype in stage II; However, in gastric cancer, the highest frequency of CC genotype is in stage I group, the highest frequency of CT genotype is in stage II and the highest frequency of TT genotype is in stage III.

In colon cancer, the highest incidence of tumor invasion was related to CC genotype in-group T2 and the lowest rate was related to group T1, but in gastric cancer, the highest frequency of tumor invasion was related to CC genotype in-group T3, and the lowest rate was related to group T1.

##### Investigating the relationship between the invasiveness and stage of the colon tumor

In colon cancer, the Spearman correlation between tumor invasiveness and tumor stage was less than 0.05 and equal to 0.000, a significant level is obtained, and the Spearman correlation value is equal to 0.507 (Table S13). Given that the Spearman correlation coefficient is positive, the relationship is direct, that is, with the increase of one of these two variables, the other increases, and vice versa.

The highest frequency is related to group T1 in stage I, and the lowest frequency is related to group T3 in stage I.

The value of the CaScore Pearson statistic is 32.224 and its significance level is 0.000, which is less than 0.05 therefore, with 0.95 confidence, the statistical null hypothesis that there is no relationship between tumor stage and tumor invasiveness is rejected. Therefore, there is a relationship between tumor stage and tumor invasiveness. T1s are mostly in the first stage; while T3s are, mostly they were placed in the third stage (Table S13).

Examining the relationship between stage and tumor invasiveness in gastric and colon cancer, we found that the highest prevalence of colon cancer was in-group T1 and in stage I but in gastric cancer in-group T3 and stage III and also in stage I but with different groups (In colon cancer T3 and stomach cancer T1) has the lowest frequency (Table S14).

By examining the relationship between tumor stage and tumor invasiveness in gastric cancer, the highest frequency is related to group T3 in stage III and the lowest frequency is related to group T1 in stage I (Table S15).

##### Investigation of gender relations with variables in colon cancer

In the study of the relationship between gender and the studied variables, the following items were found according to Table (S16, S17, S18):

1. In the study of gender and genotype, the CC genotype was 47.4% male and 52.6% female, respectively; the CT genotype comprises 55% male, and 45% female and the TT genotype was 46.3% male and 53.7% female respectively.

The value of the Chi-square-Pearson statistic is equal to 0.672 and its significance level is 0.715, which is more than 0.05, therefore, with 0.95 confidence, the statistical null hypothesis that there is no relationship between genotype and gender is confirmed, so there is no relationship between genotype and gender.

1. In examining the relationship between gender and tumor invasiveness, results such as T1 included 68.2% of men and 31.8% of women; T2 comprises 56.3% male and 43.8% female and T3 comprises 37% male and 63% female.

The value of the Chi-square-Pearson statistic is 6.54 and its significance level is 0.038, which is less than 0.05, therefore, with a confidence of 0.95, the null statistical hypothesis that there is no relationship between tumor invasiveness and gender is rejected. Therefore, there is a relationship between tumor invasiveness and gender. In the first and second stages, men are more and in the third stage, women are more.

In the Student t-test, the value of the F statistic is equal to 0.002 and its significance level is 0.968, which is more than 0.05, it shows that there is a variance for the degree of invasiveness of the tumor in terms of both male and female groups, the value of t-statistic for comparison of the two groups is equal to -2.594 and the value of probability related to its significance is equal to 0.011, which is less than 0.05, so with a confidence of 0.95, the statistical zero assumption that the mean is equal The degree of invasiveness of the tumor is rejected according to the two groups of men and women, so it can be said that there is a significant difference between the mean of men and women and the average of women is higher.

1. In the study of the relationship between gender and tumor stage, the results were as follows: 76.9% male and 23.1% female in stage I, 38% male and 62% female, 45.8% male, and 54.2% female include.

The value of the Chi-square-Pearson statistic is equal to 10.585 and its significance level is 0.005, which is less than 0.05, therefore, with a confidence of 0.95, the statistical null hypothesis that there is no relationship between tumor stage and sex is rejected. Therefore, there is a relationship between tumor stage and sex. In the first stage, men are more and in the second and third stages, women are more.

In the Student T test, the value of the F statistic is equal to 6.725 and its significance level is 0.011, which is less than 0.05 Showing that there is no variance for the tumor stage in terms of male and female groups. It is less, so with 0.95 confidence, the statistical hypothesis of zero that the mean of the tumor stage is equal in terms of both male and female groups is rejected, so it can be said that there is a significant difference between the mean of male and female groups and the average of women is higher.

With a review of colon cancer; Males have the highest frequency in CT genotype and stage I in aggressive T2 mode and females have the highest frequency in TT genotype and stage II in aggressive T3 mode.

##### Investigation of gender relations with variables in gastric cancer

In the study of the relationship between gender and the studied variables, the following items were found according to Table (S19, S20):

1. In the study of gender and genotype, the CC genotype was 74.4% male and 25.6% female, respectively; the CT genotype comprises 41.7% male, 58.3% female, and the TT genotype was 46.3% male and 53.7% female respectively.
2. In examining the relationship between gender and tumor invasiveness, results such as T1 included 47.7% of men and 52.3% of women; T2 comprises 47.7% male and 52.3% female and T3 comprises 56.4% male and 43.6% female.
3. In the study of the relationship between gender and tumor stage, the results were as follows: 56.4% male and 43.6% female in stage I, 43.6% male and 56.4% female, 52.3% male, and 47.7% female include.

There was no significant difference between the gene type of male and female patients, the tumor grade of male and female patients, and the invasive nature of the tumor in male and female patients. With a review of gastric cancer; Males have the highest frequency in the CC genotype and stage I in aggressive T3 mode and females have the highest frequency in CT genotype and stage II in aggressive T1, and T2 mode.

## Discussion

In conclusion, our results advocate that there is a link between a CDKN2A / B gene polymorphism (rs10811661) and a poor prognosis in sufferers of colorectal and gastric cancer. Humans with the TT genotype were more susceptible to colorectal and gastric cancer. Consistent with our consequences, recent research has also proven the prognostic position of CDKN2A / B in pancreatic, lung, breast, melanoma, and ovarian cancers (Qiu et al., 2015 [23]; Seifi et al., 2019 [25]; Compa et al., 2016 [40]; Schuster et al., 2014 [41].).

Those observations can be defined by way of the function of CDKN2A / B in suppressing cell proliferation and inducing tumor cellular dying. Numerous studies have proven that methylation or dysregulation of ANRIL may lessen CDKN2A / B and its downstream tumor suppressants (p14ARF and p16INK4A), which main to tumor formation and progression [42]. ANRIL has been proven to play a prime function in promoting transcriptional suppressors concerned within the discount of CDKN2A / B genes, leading to the genetic predisposition to diverse cancers (Congrains et al., 2013 [20]; Yap et al., 2010 [43]; Popov & Gil, 2010 [44]).

In keeping with this data, sun et al. ANRIL expression changed into tested in 97 tumor and non-tumor tissue samples of the CRC placenta. They found that overexpression of ANRIL in tumor tissues was related to lower survival in CRC sufferers. in addition, their laboratory results confirmed that reduction of ANRIL in CRC cell strains decreased cell proliferation and invasion (sun et al., 2016 [45]).

In another study, the correlation between ANRIL expression and clinical pathological functions of CRC became investigated in 108 sufferers. Their results indicated that overexpression of ANRIL in a CRC patient may be taken into consideration as a risk aspect for poor diagnosis and tumor metastasis (sun et al., 2016 [46]). However, the potential function of ANRIL in colorectal tumorigenesis has now not yet been decided. Recently, a huge-scale genomic correlation takes a look at was accomplished to research the association of 9p21 locus SNPs and the risk of neoplastic transformation in a couple of cancers. Their data showed that there are different genetic variations in this region related to the development of various types of cancer (Lee et al., 2014 [47]). In line with this, Gu et al. 203 analyzed SNP in the 9p21.3 area in several cancers, which include colorectal cancer. Their findings endorse that genetic variants in CDKN2A can be associated with an increased risk of colorectal cancers and other tumors (Gu et al., 2013 [24]).

Past research has shown that polymorphisms in insulin-resistant genes are effective on insulin resistance and weight gain [48, 49] as well as on the risk of developing colon cancer [50, 51]. Insulin receptors, or a decrease in insulin binding to the receptor, cause the genetic syndrome of insulin resistance [52], which in itself is a robust thing in growing the risk of developing colorectal cancer.

Ghobadi and colleagues 2019, after examining the relationship between rs10811661 and rs1333049 polymorphisms in chromosome 9 P21 locus in CDKN2A / B gene, patients with esophageal squamous cell carcinoma (ESCC) and healthy individuals concluded that in patients with carcinoma cancer, Esophageal squamous cells had a higher frequency of TT genotype for rs10811661 polymorphism than the control group and tumor size was reported to be larger in affected individuals. In addition, the mortality rate was higher in patients with CC genotype for rs1333049 polymorphism [53].

Hesari et al. 2018, in research on the 2 polymorphisms of rs1801133 in methylene tetrahydrofolate reductase and rs10811661 in CDKN2A / B in patients with breast cancers, concluded that the frequency of T allele and TT genotype in methylene tetrahydrofolate reductase gene was more prevalent in patients than in the control group. The frequency of the C allele in rs10811661’s CDKN2A / B gene was 72%. The results of the present observation are consistent with the effects of studies [54].

In a study conducted in 2018 by ShahidSales et al. On the association of rs10811661 polymorphism in CDKN2A / B in healthy and breast cancer patients, they concluded that the frequency of TT genotype was higher in breast cancer patients than in the control group. In these people, the size of the tumor is also reported to be larger. In addition, genetic studies in these individuals have shown that people with TT genotype are more likely to develop breast cancer than those with CC / CT genotypes [55].

In 2020, Rahmani et al. studied the association between rs10811661 polymorphism and colorectal cancer in 541 healthy and diseased individuals. Their research method was Taq-Man-based real-time PCR. The results confirmed the effect of this polymorphism on colorectal cancer and introduced this polymorphism as a suitable biomarker for predicting this cancer [56].

Consistent with these observations, our data support a sizeable association between CDKN2A / B gene polymorphisms, rs10811661, and colorectal and gastric cancers.

In this study, a similar result was observed, which indicates a direct relationship between TT genotype in rs10811661 polymorphism with gastric and colon cancer, tumor invasiveness, and tumor grade. In addition, there was a difference in the degree of dependence of gastric and colon cancer on the rs10811661 polymorphism genotype with sex, but the grade and aggressiveness of the tumor were not particularly dependent on the sex of the patient. Also, in different genotypes, the percentage of the frequency distribution of different phases of invasiveness and tumor grade was different, which indicates the effect of this polymorphism on these two characteristics, and also the highest frequency of tumor grade in CC grade I, in grade II CT and grade III TT and the highest percentage of T3 phase frequency, tumor invasiveness was also observed in TT condition.

### Electronic supplementary material

Below is the link to the electronic supplementary material.

Supplementary Material 1

## Data Availability

The data that support the findings of this study are available from Imam Khomeini Hospital, but restrictions apply to the availability of these data, which were used under license for the current study, and so are not publicly available. Data are however available from the authors upon reasonable request and with permission of Imam Khomeini Hospital.
