# Supplementary material for: Evaluation of rs10811661 polymorphism in CDKN2A / B in colon and gastric cancer
Source: BMC Cancer. 2023 Oct 16;23:985. doi: 10.1186/s12885-023-11461-6 (PMC10577985; doi:10.1186/s12885-023-11461-6)
Supplement: Supplementary file 1 — Supplementary Material 1 [file 12885_2023_11461_MOESM1_ESM.docx]

**Table S1- Frequency of cases studied in colon cancer**

| **Tumor invasiveness** | | | **Tumor stage** | | | **Age** | | | | **Genotype** | | |  |
| --- | --- | --- | --- | --- | --- | --- | --- | --- | --- | --- | --- | --- | --- |
| **T_3_** | **T_2_** | **T_1_** | **III stage** | **II stage** | **I stage** | **51-60** | **41-50** | **31-40** | **30<** | **TT** | **CT** | **CC** |  |
| 46 | 32 | 22 | 24 | 50 | 26 | 7 | 36 | 40 | 17 | 41 | 40 | 19 | **Frequency** |
| 46 | 32 | 22 | 24 | 50 | 26 | 7 | 36 | 40 | 40 | 41 | 40 | 19 | **Percentage of frequency** |
| 100 | 54 | 22 | 100 | 76 | 26 | 100 | 93 | 57 | 17 | NA | NA | NA | **The cumulative percentage** |

**Table S2 - Frequency of cases studied in gastric cancer**

| **Tumor invasiveness** | | | **Tumor stage** | | | **Age** | | | **Genotype** | | |  |
| --- | --- | --- | --- | --- | --- | --- | --- | --- | --- | --- | --- | --- |
| **T_3_** | **T_2_** | **T_1_** | **III stage** | **II stage** | **I stage** | **44-52** | **35-43** | **25-34** | **TT** | **CT** | **CC** |  |
| 46 | 32 | 22 | 31 | 37 | 32 | 32 | 37 | .31 | 41 | 40 | 19 | **Frequency** |
| 46 | 32 | 22 | 31 | 37 | 32 | 32 | 37 | 31 | 41 | 40 | 19 | **Percentage of frequency** |
| 100 | 54 | 22 | 100 | 69 | 32 | 100 | 68 | 31 | NA | NA | NA | **The cumulative percentage** |

**Table S3- Significant examination of Spearman correlation hypothesis in colon cancer**

| **Type** | **The correlation** | **Sample size** | **meaningful** |
| --- | --- | --- | --- |
| **genotype** | -0.123 | 100 | 0.223 |
| **Tumor invasiveness** | -0.006 | 100 | 0.955 |
| **tumor stage** | -0.12 | 100 | 0.232 |

**Table S4 -ANOVA analysis of variance in colon cancer**

|  | **genotype** | | **Tumor invasiveness** | | **tumor stage** | |
| --- | --- | --- | --- | --- | --- | --- |
|  | **Out of group** | **Intergroup** | **Out of group** | **Intergroup** | **Out of group** | **Intergroup** |
| **Total squares** | 99.711 | 5777.449 | 7.006 | 5870.154 | 91.469 | 5785/691 |
| **Degrees of freedom** | 2 | 97 | 2 | 97 | 2 | 97 |
| **Average squares** | 49.855 | 59.561 | 3.503 | 60.517 | 45.734 | 59.646 |
| **F** | 0.837 | NA | 0.058 | NA | 0.767 | NA |
| **The significance level** | 0.436 | NA | 0.944 | NA | 0.467 | NA |

**Table S5 - Average age in terms of variables in colon cancer**

|  | **genotype** | | | **Tumor invasiveness** | | | **tumor stage** | | |
| --- | --- | --- | --- | --- | --- | --- | --- | --- | --- |
|  | **CC** | **CT** | **TT** | **T_1_** | **T_2_** | **T_3_** | **I** | **II** | **III** |
| **Sample size** | 19 | 40 | 41 | 22 | 32 | 46 | 26 | 50 | 24 |
| **Average** | 40.7368 | 39.625 | 38.122 | 39.5 | 38.8438 | 39.3478 | 40.5769 | 39.16 | 37.875 |
| **Standard deviation** | 9.01753 | 7.69884 | 7.07529 | 7.60795 | 8.00338 | 7.56098 | 7.5428 | 7.49247 | 8.37342 |

**Table S6 - Significant examination of Spearman correlation hypothesis in gastric cancer**

| **Type** | **The correlation** | **Sample size** | **meaningful** |
| --- | --- | --- | --- |
| **genotype** | -0.123 | 100 | 0.223 |
| **Tumor invasiveness** | 0.108 | 100 | 0.285 |
| **tumor stage** | -0.32 | 100 | 0.755 |

**Table S7- ANOVA analysis of variance in gastric cancer**

|  | **genotype** | | **Tumor invasiveness** | | **tumor stage** | |
| --- | --- | --- | --- | --- | --- | --- |
|  | **Out of group** | **Intergroup** | **Out of group** | **Intergroup** | **Out of group** | **Intergroup** |
| **Total squares** | 99.711 | 5777.449 | 7.006 | 5870.154 | 91.469 | 5785/691 |
| **Degrees of freedom** | 2 | 97 | 2 | 97 | 2 | 97 |
| **Average squares** | 49.855 | 59.561 | 3.503 | 60.517 | 45.734 | 59.646 |
| **F** | 0.837 | NA | 0.058 | NA | 0.767 | NA |
| **The significance level** | 0.436 | NA | 0.944 | NA | 0.467 | NA |

**Table S8 - Average age in terms of variables in gastric cancer**

|  | **genotype** | | | **Tumor invasiveness** | | | **tumor stage** | | |
| --- | --- | --- | --- | --- | --- | --- | --- | --- | --- |
|  | **CC** | **CT** | **TT** | **T_1_** | **T_2_** | **T_3_** | **I** | **II** | **III** |
| **Sample size** | 19 | 40 | 41 | 22 | 32 | 46 | 32 | 37 | 31 |
| **Average** | 40.7368 | 39.625 | 38.122 | 39.5 | 38.8438 | 39.3478 | 40.5769 | 39.16 | 37.875 |
| **Standard deviation** | 9.01753 | 7.69884 | 7.07529 | 7.60795 | 8.00338 | 7.56098 | 7.5428 | 7.49247 | 8.37342 |

**Table S9 - Descriptive statistics to investigate the relationship between tumor stage and genotype in colon cancer**

| **Genotype types** | **Tumor stage** | | | **Tumor invasiveness** | | |
| --- | --- | --- | --- | --- | --- | --- |
|  | **I** | **II** | **III** | **T_1_** | **T_2_** | **T_3_** |
| **CC** | 6  23.10% | 12  46.20% | 8  30.8% | 3  13.6% | 11  50% | 8  36.4% |
| **CT** | 9  18% | 17  34% | 24  48% | 6  18/8% | 13  40.6% | 13  40.6% |
| **TT** | 4  16.7% | 11  45.8% | 9  37.5% | 10  21.7% | 16  34.8% | 20  43.5% |

**Table S10 - Chi-square test in colon cancer**

|  | **Tumor stage** | | | **Tumor invasiveness** | | |
| --- | --- | --- | --- | --- | --- | --- |
|  | **Value** | **Degrees of freedom** | **Meaningful level** | **Value** | **Degrees of freedom** | **Meaningful level** |
| **Chi Square Pearson** | 2.556 | 4 | 0.635 | 1.567 | 4 | 0.815 |
| **Probability ratio** | 2.582 | 4 | 0.63 | 1.573 | 4 | 0.814 |
| **Fisher's exact test** | 2.63 | NA | 0.638 | 1.539 | NA | 0.836 |
| **Line by line** | 0.425 | 1 | 0.514 | 0.002 | 1 | 0.962 |
| **Sample size** | 100 | NA | NA | 100 | NA | NA |

**Table S11 - Kruskal-Walli’s test in colon cancer**

|  | **Tumor stage** | **Tumor invasiveness** |
| --- | --- | --- |
| **Amara Kai Do.** | 0.447 | 1.461 |
| **Degrees of freedom** | 2 | 2 |
| **Meaningful level** | 0.8 | 0.482 |

**Table S12 - Descriptive statistics to investigate the relationship between tumor stage and genotype in gastric cancer**

| **Genotype types** | **Tumor stage** | | | **Tumor invasiveness** | | |
| --- | --- | --- | --- | --- | --- | --- |
|  | **I** | **II** | **III** | **T_1_** | **T_2_** | **T_3_** |
| **CC** | 10  64.10% | 4  17.62% | 5  18.28% | 2  13.6% | 8  42.9% | 9  43.5% |
| **CT** | 12  19% | 18  58% | 10  23% | 11  15% | 16  44.4% | 13  40.6% |
| **TT** | 10  17% | 15  41% | 16  42% | 9  18.2% | 8  16.6% | 24  65.2% |

**Table S13 - Chi-square test to assess the stage of the tumor and the degree of invasiveness of the colon tumor**

|  | **Value** | **Degrees of freedom** | **Meaningful level** |
| --- | --- | --- | --- |
| **Chi Square Pearson** | 29.336 | 4 | 0.000 |
| **Probability ratio** | 34.547 | 4 | 0.000 |
| **Fisher's exact test** | 32.224 | NA | 0.000 |
| **Line by line** | 24.042 | 1 | 0.000 |
| **Sample size** | 100 | NA | NA |

**Table S14 -Descriptive statistics of the relationship between being aggressive and the stage of the colon tumor**

|  | **T_1_** | **T_2_** | **T_3_** |
| --- | --- | --- | --- |
| **Stage I.** | 13  54.50% | 12  40.60% | 1  2.20% |
| **Stage II.** | 8  36.40% | 15  46.90% | 27  58.70 |
| **Stage III** | 2  9.10 | 4  12.50 | 18  39.10 |

**Table S15 - Descriptive statistics of the relationship between being aggressive and the stage of the gastric tumor**

|  | **T_1_** | **T_2_** | **T_3_** |
| --- | --- | --- | --- |
| **Stage I.** | 6  10.8% | 13  44.6% | 13  44.6% |
| **Stage II.** | 9  20.65% | 9  20.65% | 19  58.70% |
| **Stage III** | 7  27.3% | 10  35.4% | 14  40.9% |

**Table S16 - Descriptive statistics on the relationship between gender and the variables studied in colon cancer**

|  | **Genotype** | | | **Tumor stage** | | | **Tumor invasiveness** | | |
| --- | --- | --- | --- | --- | --- | --- | --- | --- | --- |
|  | **CC** | **CT** | **TT** | **I** | **II** | **III** | **T_1_** | **T_2_** | **T_3_** |
| **Man** | 9  47.4% | 22  55% | 19  46.30% | 20  76.90% | 19  38% | 11  45.80% | 15  68.20% | 18  56.30% | 17  37% |
| **Female** | 10  52.6% | 18  45% | 22  53.7% | 6  23.1% | 31  62% | 13  54.20% | 7  31.8% | 14  43.8% | 29  63% |

**Table S17- Chi-square test of the relationship between gender and the variables studied in colon cancer**

|  | **Tumor stage** | | | **Tumor invasiveness** | | | **Genotype** | | |
| --- | --- | --- | --- | --- | --- | --- | --- | --- | --- |
|  | **Value** | **Degrees of freedom** | **Meaningful level** | **Value** | **Degrees of freedom** | **Meaningful level** | **Value** | **Degrees of freedom** | **Meaningful level** |
| **Chi Square Pearson** | 10.585 | 2 | 0.005 | 6.54 | 2 | 0.038 | 0.672 | 2 | 0.715 |
| **Probability ratio** | 11.028 | 2 | 0.004 | 6.645 | 2 | 0.036 | 0.673 | 2 | 0.714 |
| **Line by line** | 5.073 | 1 | 0.024 | 6.362 | 1 | 0.012 | 0.072 | 1 | 0.789 |
| **Sample size** | 100 | NA | NA | 100 | NA | NA | 100 | NA | NA |

**Table S18- Student's t-test examining variables by gender in colon cancer**

|  | **Tumor stage** | **Tumor invasiveness** |
| --- | --- | --- |
| **F** | 6.725 | 0.002 |
| **Meaningful level** | 0.011 | 0.968 |
| **t** | -2.301 | -2.594 |
|  | -2.301 | -2.594 |
| **Degrees of freedom** | 98 | 98 |
|  | 92.677 | 97.102 |
| **Meaningful level** | 0.024 | 0.011 |
|  | 0.024 | 0.011 |

**Table S19- Descriptive statistics on the relationship between gender and the variables studied in gastric cancer**

|  | **Genotype** | | | **Tumor stage** | | | **Tumor invasiveness** | | |
| --- | --- | --- | --- | --- | --- | --- | --- | --- | --- |
|  | **CC** | **CT** | **TT** | **I** | **II** | **III** | **T_1_** | **T_2_** | **T_3_** |
| **Man** | 20  74.4% | 22  41.7% | 8  46.30% | 18  56.4% | 16  43.6% | 16  52.3% | 10  47.7% | 15  47.7% | 25  56.4% |
| **Female** | 12  25.6% | 28  58.3% | 10  53.7% | 14  43.6% | 21  56.4% | 15  47.7% | 12  52.3% | 17  52.3% | 21  43.6% |

**Table S20- Statistics and analysis of variance of studied treatments in gastric cancer**

|  | | **Genotype** | | **Tumor stage** | | **Tumor invasiveness** | |
| --- | --- | --- | --- | --- | --- | --- | --- |
|  |  | **Man** | **Female** | **Man** | **Female** | **Man** | **Female** |
| **Amara Loon** | **Statistical average coefficients** | 2.02 | 2.4 | 1.96 | 2.02 | 2.30 | 2.18 |
|  | **Meaningful level** | 0.510 | 0.510 | 0.276 | 0.276 | 0.881 | 0.881 |
| **T-test for equality of means** | **T-test** | -2.634 | -2.634 | -0.374 | -0.374 | 0.775 | 0.775 |
|  | **Degrees of freedom** | 98 | 98 | 98 | 98 | 98 | 98 |
|  | **The significance level for the two domains** | 0.10 | 0.10 | 0.709 | 0.709 | 0.452 | 0.452 |
|  | **Mean difference** | -0.380 | -0.380 | -0.060 | -0.060 | 0.120 | 0.120 |


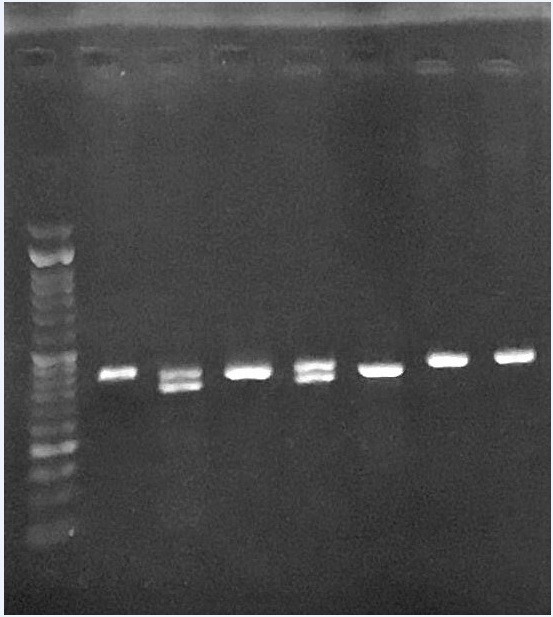


Figure 1: The first column on the right is for DNA-sized markers. The second column is related to the homozygous genotype (CC) with a 393 bp bond. The third column represents the homozygous genotype (TT) with 344 and 49 bp bonds.
